# Development of psychiatric risk evaluation checklist and routine for nurses in a general hospital: ethnographic qualitative study: Desenvolvimento de *check-list* e rotina de avaliação de risco psiquiátrico para enfermeiras num hospital geral: pesquisa qualitativa etnográfica

**DOI:** 10.1590/1516-3180.2013.8100711

**Published:** 2014-11-28

**Authors:** Ana Luiza Lourenço Simões Camargo, Alfredo Maluf, Fátima Tahira Colman, Vanessa de Albuquerque Citero

**Affiliations:** I MD, MSc. Doctoral Student, Department of Psychiatry, Escola Paulista de Medicina - Universidade Federal de São Paulo (EPM-Unifesp), and Medical Coordinator of the Department of Psychosomatic Medicine and Psychiatry, Hospital Israelita Albert Einstein (HIAE), São Paulo, Brazil.; II MD. Psychiatrist, Department of Psychosomatic Medicine and Psychiatry, Hospital Israelita Albert Einstein (HIAE), São Paulo, Brazil.; III RN. Manager, Inpatient Department, Hospital Israelita Albert Einstein (HIAE), São Paulo, Brazil.; IV MD, PhD. Affiliated Professor, Department of Psychiatry, Escola Paulista de Medicina - Universidade Federal de Sao Paulo (EPM-Unifesp), São Paulo, Brazil.

**Keywords:** Psychosomatic medicine, Nurses, Psychiatry, Hospitals, general, Risk assessment, Medicina psicossomática, Enfermeiras, Psiquiatria, Hospitais gerais, Medição de risco

## Abstract

**CONTEXT AND OBJECTIVE:**

There is high prevalence of mental and behavioral disorders in general hospitals, thus triggering psychiatric risk situations. This study aimed to develop a psychiatric risk assessment checklist and routine for nurses, the Psychiatric Risk Evaluation Check-List (PRE-CL), as an alternative model for early identification and management of these situations in general hospitals.

**DESIGN AND SETTING:**

Ethnographic qualitative study in a tertiary-level private hospital.

**METHOD:**

Three hundred general-unit nurses participated in the study. Reports were gathered through open groups conducted by a trained nurse, at shift changes for two months. The questions used were: “Would you consider it helpful to discuss daily practice situations with a psychiatrist? Which situations?” The data were qualitatively analyzed through an ethnographic approach.

**RESULTS:**

The nurses considered it useful to discuss daily practice situations relating to mental and behavioral disorders with a psychiatrist. Their reports were used to develop PRE-CL, within the patient overall risk assessment routine for all inpatients within 24 hours after admission and every 48 hours thereafter. Whenever one item was present, the psychosomatic medicine team was notified. They went to the unit, gathered data from the nurses, patient files and, if necessary, attending doctors, and decided on the risk management: guidance, safety measures or mental health consultation.

**CONCLUSION:**

It is possible to develop a model for detecting and intervening in psychiatric and behavioral disorders at general hospitals based on nursing team observations, through a checklist that takes these observations into account and a routine inserted into daily practice.

## RESUMO

**CONTEXTO E OBJETIVO:**

Existe alta prevalência de transtornos mentais e comportamentais em hospitais gerais, propiciando situações de risco psiquiátrico. Este estudo objetivou desenvolver uma rotina e um *check-list* para enfermeiras, a Avaliação de Risco Psiquiátrico (ARP-CL), como modelo alternativo de identificação e manejo precoce destas situações no hospital geral.

**TIPO DE ESTUDO E LOCAL:**

Pesquisa qualitativa etnográfica, em hospital particular terciário.

**MÉTODO:**

Trezentas enfermeiras de unidades gerais participaram do estudo. Os relatos foram coletados em grupos abertos, conduzidos por enfermeira treinada, durante passagens de plantão, por dois meses, através das questões: “Você consideraria útil discutir com um psiquiatra situações da sua prática diária? Quais situações?” Os dados foram analisados qualitativamente através do método etnográfico.

**RESULTADOS:**

Enfermeiras consideraram útil poder discutir rotineiramente com um psiquiatra situações relacionadas a transtornos mentais e de comportamento da sua prática diária. Seus relatos foram utilizados no desenvolvimento da ARP-CL, na rotina da avaliação de risco global do paciente, para todos os internados nas primeiras 24 horas e posteriormente a cada 48 horas. Quando um item era presente, a equipe de medicina psicossomática era notificada, indo à ala e coletando dados com a enfermagem, no prontuário do paciente, ou com o médico assistente, se necessário, decidindo conduta no risco: orientação, medidas de segurança ou consulta em saúde mental.

**CONCLUSÃO:**

É possível desenvolver um modelo de detecção e intervenção precoces para transtornos psiquiátricos e de comportamento num hospital geral baseado na observação de enfermeiras, através de *check-list* que leve em conta essas observações e de uma rotina inserida na prática diária.

## INTRODUCTION

There is high prevalence of mental and behavioral disorders in general hospitals.^1^^,^^2^^,^^3^^,^^4^ Psychiatric consultation-liaison services provide the classic model for detection, management and care in these settings,^5^ potentially enhancing the quality of care, safety measures and teaching opportunities concerning mental health.^5^^,^^6^ Nevertheless, they require economic investments that are not always attractive, given that there is no proof that they contribute towards hospitals’ financial earnings.^7^

In Brazil, there are few structured consultation-liaison services in private, non-governmental general hospitals. This reality implies that, in these settings, patients in need of psychiatric care depend on their private doctors to identify emotional distress and trigger psychiatric consultations, which are usually conducted by private-practice psychiatrists and depend either on the patient’s own means or on their health insurance coverage. Furthermore, such services sometimes do not provide enough information for healthcare teams regarding how to manage behavioral and/or emotional situations that occur among patients in general wards.

Surprisingly, even in the presence of structured consultation-liaison services, only 1% to 13% of patients admitted to general hospitals are referred to specialists.^1^^,^^4^ This small number of referrals may be associated with low rates of detection of psychiatric disorders by physicians and nurses,^8^ either due to lack of knowledge or due to difficulty in differentiating these symptoms from those of clinical and surgical diseases.^3^^,^^9^ Patients with behavioral disorders who are not referred to specialists are often subject to insufficient attention and care, in addition to inadequate psychiatric treatment,^1^^,^^10^ thereby leading to psychiatric risk situations.

The epidemiological concept of “risk” implies that events with unfavorable outcomes may occur.^11^ There are clinical, social and economic risks involved in psychiatric events,^12^ such as psychological distress and psychiatric disorders relating to worse prognosis for clinical diseases;^4^^,^^13^^,^^14^^,^^15^^,^^16^^,^^17^ behavioral changes with an impact on clinical treatment during hospital stay;^2^ low detection, by the healthcare team, of mental disorders and self-harming behavior or suicidal ideation;^1^^,^^18^^,^^19^ non-accurate psychiatric diagnoses;^1^^,^^20^ inappropriate treatment or undertreatment of mental disorders;^21^^,^^22^ and admission of patients with psychiatric disorders to clinical-surgical units without proper support.^23^

## OBJECTIVE

Using the concept of risk as a possible screener for psychiatric and behavioral disorders or mental distress, the present study aimed to describe the development of a psychiatric risk assessment tool, the Psychiatric Risk Evaluation Checklist (PRE-CL), and a model for a psychiatric risk assessment routine, in order to provide a different model for early identification and appropriate management of psychiatric risk in general hospitals, taking into account institutional safety and quality-of-care goals.

## METHODS

The PRE-CL was developed in an ethnographic study that was conducted in 2004. The setting was a 512-bed private general-care non-profit hospital, located in São Paulo, Brazil, with medical care delivered not only by the hospital’s medical staff (emergency department, intensive care and step-down unit and coronary, obstetrics, nursery, dialysis and rehabilitation units), but also by private-practice doctors who admit their patients to the hospital and decide on their treatment. At that time, the hospital did not have a specific unit for mental disorders, and it was an institutional policy not to admit patients in need of exclusively psychiatric treatment.

However, the hospital’s medical practice division became highly concerned about events relating to patients who had been admitted for clinical-surgical treatment and who presented either psychiatric or severe behavioral disorders during their stay: patients leaving hospital without medical consent; attempting to use narcotic substances in the hospital’s facilities; presenting suicidal thoughts without psychiatric evaluation; or exhibiting disruptive behavior inside the hospital. Moreover, there were frequent occurrences of inappropriate medical prescriptions of narcotics and admissions of patients with exogenous intoxications into the clinical-surgical units without psychiatric monitoring.

These high-risk events, whenever they happened and no matter how frequent they were, compromised quality of care and patient safety (as well as family, visitor and staff safety), and required mobilization of a great quantity of resources from the institution as a whole. With the aim of developing a care model that would allow the healthcare staff to promptly identify risk situations relating to inpatients’ psychiatric or severe behavioral disorders, thereby providing early and adequate interventions, Hospital Israelita Albert Einstein (HIAE) created a Department of Psychosomatic Medicine composed of two doctors: a consultation-liaison psychiatrist and an addiction specialist. It was decided that the new system should work using the risk concept applied to psychiatric issues and should be included in the nurse’s overall risk assessment,^18^ which is a tool used by the nursing team to evaluate different types of risk during hospitalization (nutritional risk, social risk, need for physiotherapeutic care, psychological risk, risk of falls, need for occupational therapeutic care, phlebitis and pain).

Registered nurses are healthcare professionals who are present in all clinical and surgical units 24 hours a day. Hence, they have an important role in potentially identifying risk situations that should be reassessed continuously. On the other hand, such findings should be systematically discussed with the psychiatric team. Since it had been decided that nurses would be the ones to use the risk identification tool, it became essential that this tool should be user-friendly and coherent with nurses’ beliefs about psychiatric risks, dissonant behavior and the help that discussion of cases with a psychiatrist would provide in their daily routine, so that they would be able to adhere to the future routine.

For situations to be identified as presenting risks, they have to be regarded as such by the individuals involved in their recognition.^24^ Therefore, a qualitative ethnographic survey of the hospital’s nursing population was carried out, after obtaining approval from the hospital’s Ethics Committee.

The methodology chosen allowed the researchers to learn about the impressions of this population based on its specific cultural context^24^^,^^25^^,^^26^ and, later on, to prepare the checklist. This survey aimed to understand the following questions:

•Did the hospital’s nurses think that it would be useful to have a psychiatrist in their units?
- In which situations did nurses find a psychiatrist useful?
- Did these situations agree with the proposal for psychiatric inter-consulting and support in the general hospital?

It would also be important to observe the terminology used by the nursing staff in describing these situations, with the aim of designing an assessment tool for the psychiatric risks to be reported by this nursing population.

The participants in the survey included the 300 registered nurses who were on duty in clinical units (cardiology, gastroenterology, neurology, pneumology and internal medicine), as well as in surgery, geriatrics, pediatrics, oncology, the maternity ward, intensive care units, step-down units, gynecology and obstetrics and the day clinic. Over a two-month period (April and May 2004), reports were gathered through open groups, conducted by a trained nurse at shift changes (three times per 24 hours: morning, afternoon and night), with participation of three to four registered nurses per group and with two groups in each unit. These were repeated at least twice in all inpatient units, in each shift.

The nurses were invited to briefly interrupt their shift change discussions and talk to the trained nurse, and no refusals to participate were observed. The reports were manually recorded so that situations in which the participants might feel intimidated by a tape recorder could be avoided. To initially approach the group, two questions were asked: 1. “Would you consider it helpful to be able to discuss daily practice situations with a psychiatrist?” 2. “In which situations?”

The knowledge that there was a sense of need for a psychiatric approach to situations that were present in the nurses’ daily practice was very important, since we believed that their adherence to the protocol would only be possible if it had a correspondence to their needs. Nevertheless, it would be important for the reported situations to be congruent with the scope of the patients’ intervention checklist and not to represent other interests, such as personal needs.

The reports were qualitatively analyzed using an ethnographic approach, with recurrent themes grouped into categories that represented the cultural point of view of the study population.^25^

## RESULTS

“...what is simple becomes a problem ...” (report from a nurse).

We observed that the nurses’ reports acknowledged the usefulness of having discussions with a psychiatrist about situations relating to mental and behavioral disorders that they identified in their clinical practice, as shown below:

- Psychiatric diagnosis - histories of mental disorders without specialized monitoring during hospital stay, strange behaviors without diagnosis, symptoms observed during stay and admission of psychiatric cases uncovered by alleged medical conditions.
- Psychiatric treatment - absence of treatment for patients with behavioral changes and multi-medication without diagnosis or opinion from specialist.
- Behavioral changes affecting medical treatment - apathy, aggressiveness and fear.
- Risks to the staff and patient - agitation and aggressiveness involving both self-harm and aggressive behavior towards other people.

Their reports reflected situations that have consistently been related to demands for psychiatric monitoring, both in the scientific literature and in our hospital’s psychiatric consultation-liaison experience. Nevertheless, although the reports showed that the nursing staff perceived altered mental and behavioral functioning in patients, the terms that they used to describe what they saw (“depressed,” “confused”, etc.) were not attempts to make a psychiatric diagnosis but, rather, attempts to name a behavioral alteration that they had identified. Table 1 shows the nurses’ reports, the categories developed based on these reports and the risk items developed according to the categories.

**Table 1.**
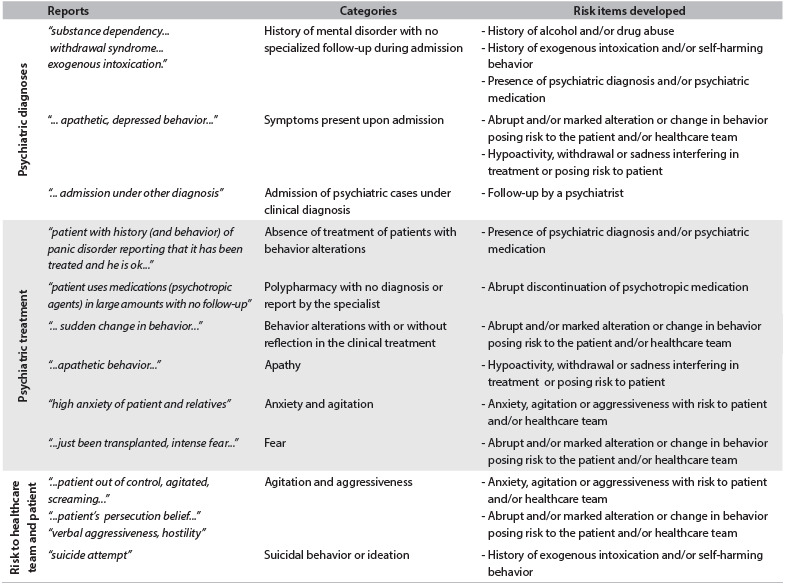
Examples of nurses’ reports, categories developed based on these reports and risk items developed according to the categories

Through these observations, it was understood that the tool should take into account these professionals’ perceptions of the situations present in their daily practice and their own way of expressing the mental and behavioral phenomena that they observed, together with general hospital needs relating to mental health, as supported by the scientific literature on consultation-liaison. For instance, suicidal behavior is a recognized risk in general hospitals, and it was represented in our tool by the expression “self-harm”, since this was more representative of nurses’ way of describing this behavior. Some of the items, especially those representing behavioral conditions, received the description “posing risk to patient and/or health team”, in an attempt to give nurses a subjective clue about which patients they could deal with and which patients presented behavioral problems of a magnitude that at least showed a need for discussion with the consultation-liaison psychiatrist.

**Chart 1.**
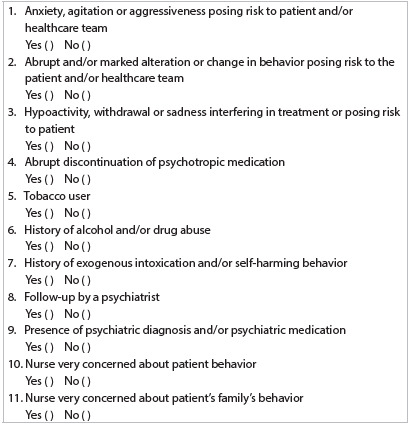
Psychiatric risk evaluation checklist

The psychiatric risk instrument that was developed is displayed in Chart 1. The first three items (1 to 3) correspond to behavioral change risks identified on a daily basis by the nursing staff, according to the results from the survey. Items 5 to 9 correspond to active observation of medical situations that have been described in the literature relating to psychiatric risk and disruptive behavior in general hospitals. Items 5, 10 and 11 were included later on, in order to encompass tobacco users (who had not been recognized by the nurses as having histories of drug use and therefore were not included in item 6), family behavioral risk situations and notification of risk whenever they subjectively felt that something was wrong but could not include their impressions in any other item.

A psychiatric risk evaluation routine was developed before officially including the PRE-CL as a part of the patient overall risk assessment, through a pilot study that was conducted in the neurology unit for two months in 2005. The aim of this was to evaluate the ease of use of the routine and the nursing team’s compliance with it. This procedure showed that the routine did not require any modifications.

In July 2005, all the hospital’s nurses were trained through 15-minute explanations with audiovisual material, during shift changes, in all units of the hospital. Simultaneously, the medical staff was informed about the new routine by means of discussion forums and specialty meetings. In August 2005, the PRE-CL was definitively included in the patient overall risk assessment routine, therefore implying that nurses would apply the tool to every inpatient within the first 24 hours after admission and would repeat the application every 48 hours during the hospital stay, or at any time when a risk situation might occur.

When at least one risk item was identified as present, the psychiatric risk was notified to the team of the Department of Psychosomatic Medicine and Psychiatry through e-mail, and this team would make a risk assessment within 24 hours. If the nurses felt that a situation might evolve to an event within a short period of time, they could also call the specialist team at any time to discuss the case and start the risk management. Once a risk had been notified, the psychiatrist went to the unit and gathered data from the nursing team about the patient and the risk situation observed, analyzed the patient record (documents, initial assessment of the patient, admission, nursing progression notes, medical progression notes and medical prescription) and, if necessary, the attending medical team was contacted for further information. The risk assessment did not add any extra costs for the payers, since it did not include a clinical evaluation of the patient.

Based on this analysis, the psychiatrist decided on the risk management, gave guidance to the nurses and, if required, the medical team as well, and this procedure was recorded in the patient’s files. The psychiatrist’s evaluation of the risk situation was filed in a database. If the evaluating psychiatrist considered a psychiatric consultation with the patient to be necessary, this suggestion would be made to the attending physician, who would be in charge of addressing the patient or the family, in order to make a referral to a psychiatrist. Once the risk had been identified and confirmed by the psychiatric team, they would monitor the situation until the risk situation ceased to exist or the patient was discharged from hospital. The psychiatrist’s risk evaluation interventions, grouped in categories and examples, are shown in Table 2.

**Table 2.**
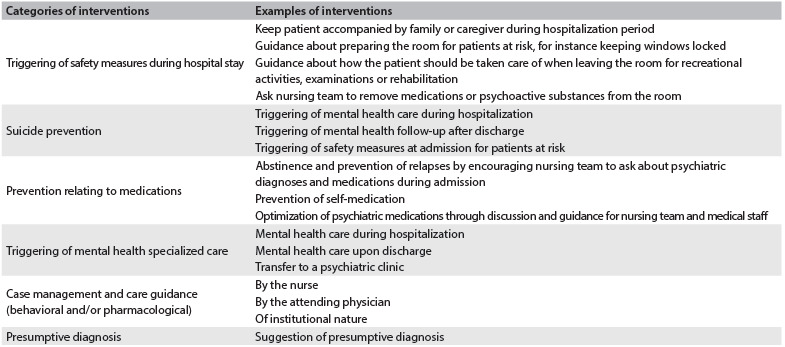
Categories and examples of risk evaluation interventions

## DISCUSSION

Patients with behavioral and/or psychiatric disorders are expected to be a part of the daily routine in clinical-surgical wards. In order to deliver proper psychiatric care in general private hospitals, streamlined models of consultation-liaison services should be implemented.^27^ Nevertheless, there are problems to be considered. Psychiatric consultations usually depend on the health team’s recognition that something is going wrong and that it should at least be discussed with a specialist. Unfortunately, the cases referred for consultation are not always those in need, and patients that would require specialized care are often not identified.^28^

The liaison model of intervention has the potential to deal with the mental health denominator, although requiring a large number of professionals present in the wards and participating in health team discussions, and thus resulting in quite an expensive and almost prohibitive way of delivering psychiatric care.^2^ Hence, having a tool that potentially triggers discussions with the specialist and an institutional work flow that detects, evaluates and manages situations that are considered to present psychiatric risk may represent a way of preserving mental health, quality of care, teaching and safety in clinical-surgical wards, with a small number of specialists and therefore at lower cost.

The existence of a psychiatric risk evaluation routine inserted into nurses’ daily activities may lead to discussions and interventions even in cases that would not normally be referred for psychiatric consultation. Through this routine, the healthcare team can be supported by the specialist, and their observations about behavioral phenomena can be taken in account. When something feels wrong, despite knowing exactly what is happening, nurses can still use the routine to trigger a risk evaluation, so that they are able to discuss situations in which otherwise fear of the appearance of impropriety could have inhibited further discussion or intervention. In this sense, it is possible that the risk evaluation routine can contribute towards changing the healthcare team’s attitudes and paradigms relating to mental diseases, thus providing a positive and illuminating experience through information and support.^5^^,^^29^

Interestingly, emergency situations are sometimes brought by phone to the psychosomatic medicine team even before the risk is notified. This is a pattern that we observe frequently in the liaison model, with the feeling that the psychiatrist is part of the health team and will be there to help when needed. The fact that it is nurses’ daily obligation to assess psychiatric risk among other clinical risks, may bring some seriousness into this matter: potentially, mental health becomes a subject to be taken in account when it comes to quality of care and patients’ security, just like any other clinical condition. Moreover, perhaps through providing systemization of what to pay attention to, the PRE-CL can enhance the idea that mental issues do exist and need to be addressed. Nurses are usually the healthcare team members that are always near the patients, and they centralize the clinical information and care routines.

Through this study, we observed that nurses seem to be aware of behavioral risk situations. It is possible that having a psychiatric risk evaluation routine that is not only part of their daily obligations but also supported by the hospital board can reinforce their belief in what they see and enhance their adherence to this routine.

It is also possible that none of this would happen if the PRE-CL was not user-friendly or meaningful to the nursing staff.^24^ That is why we considered it important to proceed with an ethnographic study in order to provide information about how nurses saw mental health features.

It is a peculiarity of our hospital that consulting psychiatrists are often private-practice doctors, who are not always familiar with hospital routines and security actions and not used to providing the nursing team with proper information on the patient’s mental health care needs in clinical-surgical wards. Also, sometimes, even if the patient is being followed up at home or within primary care by a psychiatrist, he/she often does not agree to attend this patient during the hospital stay for clinical or surgical reasons.

Therefore, clinicians and surgeons often take charge of treating their patients’ comorbid psychiatric disorders. In this regard, the evaluation routine allows discussion and support for situations that can be dealt with by general doctors with the aid of the specialist, or triggers mental health consultations in extreme cases. Further studies are necessary in order to assess whether there are any risk situations that are not detected by the nursing team and whether lack of detection, if present, reflects a failure in our tool. One important factor is that the tool can always be renewed, so as to take into account the population that fills out the data form and the peculiarities of the hospital population, in addition to structural changes in the institution. Through the PRE-CL, we also have the possibility of becoming acquainted with what the institution’s needs are and how they might change through time. One example of this is that we observed that some risks started to be notified based on the behavior of family members. This showed that there was a need to work with this population, and we were subsequently able to include a specific item in the PRE-CL to address behavioral features observed in family members. This was not surprising, because the presence of relatives during hospital admissions is a strong feature in Brazil,^30^ but it shows how cultural differences should be taken into consideration when building such a tool and routine.

Once again, the information provided by the protocol itself showed the way in which it should be dynamically and constantly adjusted to the institutional needs. One interesting fact to bear in mind is that one of the PRE-CL items focuses on suicidal behavior, which is a major risk that should be addressed systematically among inpatients in general hospitals, since suicide in hospital settings and after discharge are major issues in mental health and safety.^1^^,^^18^^,^^19^

It is possible that development of a care model for psycho-somatic medicine, based precisely on the concept of risk, which is usually a major concern in private general hospitals, may call for a deeper look at mental health demands, thereby fostering long-needed actions, such as:

- Development of further research on new models for care relating to general hospital psychiatry.
- Development of institutional routines to deal with behavioral and psychiatric disorders in clinical-surgical inpatient units.
- Provision of proper information on mental health disorders for healthcare teams.
- Training for healthcare teams with regard to early detection and management of behavioral disorders.
- Changes to mental health paradigms in general hospitals.

Concerning further research on PRE-CL and its routines, validation and reliability are important issues that need to be addressed. Studies are already being developed in order to identify at-risk populations and understand how nurses identify the severity of the risks, and how risk interventions may interfere with care during the hospital stay.

### Clinical implications

We observed that the psychiatric risk evaluation routine led to several changes to the paradigms regarding psychiatric care for clinical and surgical patients at our service. De Albuquerque Citero et al.^30^ suggested that this could be a parameter for the importance of psychiatric actions at general hospitals. For example: in the beginning, it was observed that physicians did not want to have a specialist discussing their patients with the nursing team, or even reading the patients’ files. Implementation of the psychiatric risk assessment gradually changed this approach and, today, physicians themselves often ask the nursing staff to evaluate and notify the psychiatric risk in order to receive guidance on how to manage the case. The routine has also provided the Department of Psychosomatic Medicine with the opportunity to map out the profile of the institution’s patients according to mental health issues and therefore to propose more appropriate interventions with optimization of economic resources, on the basis of documented facts. By acknowledging the needs relating to quality and safety, it was possible to discuss them at the institutional level and develop care protocols for agitated and aggressive patients and safety routines for admissions, as well as an inpatient unit based on the concept of psychosomatic medicine. In this unit, the clinical nursing staff, which has been trained on management of behavioral disorders through the psychiatric risk evaluation routine, gives support to clinical and surgical patients with behavioral disorders who demand specialized care, as well as to patients with psychiatric disorders who are voluntarily hospitalized. After a request from the nursing staff, the use of the PRE-CL was expanded to the chemotherapy, radiotherapy and rehabilitation outpatient clinics. We were able to apply the routine in these settings with very few changes, which, once again, showed us the versatility of this care model.

## CONCLUSION

It is possible to develop a model for detecting and intervening in psychiatric and behavioral disorders at general hospitals based on observations made by the nursing team, by means of a checklist that takes into account the way in which these professionals describe and/or report these behaviors, and by means of a routine inserted into their daily practice. This instrument probably made it easier for the nurses to organize what they saw and to call the specialist without having to justify this option with a clinical diagnosis.

It is important that the instrument and the protocol should be flexible in relation to the changes in paradigms and contexts that occur at general hospitals over time, taking into consideration the healthcare team’s daily experiences and impressions about their practice.
